# High prevalence of sensitization to non-specific lipid transfer protein in adult patients with primary eosinophilic gastrointestinal disorders in Italy: a single center series

**DOI:** 10.1186/s12948-022-00174-z

**Published:** 2022-07-20

**Authors:** Carlo Maria Rossi, Marco Vincenzo Lenti, Giovanna Achilli, Stefania Merli, Aurelio Mauro, Andrea Anderloni, Antonio Di Sabatino

**Affiliations:** 1 University of Pavia, Pavia, Italy; 2 First Department of Internal Medicine, Fondazione IRCCS San Matteo, Pavia, Italy; 3 Gastrointestinal Endoscopy Unit, Fondazione IRCCS San Matteo, Pavia, Italy

**Keywords:** Diet, Eosinophilic esophagitis, Food allergy, Non-specific lipid transfer protein, Peach

Dear editor

Primary eosinophilic gastrointestinal disorders (EGID) are globally increasing in prevalence, having a high disease burden. An increased awareness of these entities has allowed -at least for some of them- an earlier diagnosis. Besides, pathogenesis-driven therapies will probably be available shortly [1, 2].

Type 2 inflammation and allergy have shown to be central to the pathogenesis of eosinophilic esophagitis (EoE) [3], as reflected by the beneficial effect of topical steroids and allergen-free diets, although the understanding of the mechanistic development of this disorder is still elusive. Inhaled and ingested allergens of various sources and allergens administered for immunotherapy, via both the sublingual and oral routes, are recognized as important triggers, acting through cellular-mediated and IgE-mediated mechanisms. The main foods deemed to be relevant in the pathogenesis are cow milk, wheat, egg, soy/legumes, seafood, and nuts. Among allergen classes, PR-10 has been found to be implicated in adults with EoE [4]. Non-specific lipid transfer protein (nsLTP) among food allergens has been given much attention due to the fact that the main studies on EoE have been carried out in the US where the sensitization to this allergen is not as relevant as in the Mediterranean area. Moreover, the pathogenesis of EGID not affecting the esophagus and the role of allergic sensitization is even unclearer [1], although an allergic component has been postulated.

On these bases, in the present study we retrospectively evaluated all consecutive cases of adult-onset primary EGID referred to our allergy outpatient clinic in Northern Italy, from 2020 to 2021, for assessing the sensitization profile to food and other allergens. The diagnosis of primary EGID was made according to internationally-recognized criteria, including tissue eosinophil infiltration reaching the predefined cut-off level for each segment of the gastrointestinal tract, after the exclusion of secondary or systemic causes, such as drug reactions, helminthic infections, and hypereosinophilic syndrome [2].

After a careful history taking, including ongoing therapies for EGID, patients underwent a comprehensive screening of allergic comorbidities (i.e., atopic dermatitis, allergic rhinitis, asthma, and allergies to inhalants, food, drugs and *Hymenoptera* venom). Skin prick tests for inhalants and food allergens (Lofarma, Italy, and ALK, Denmark) were carried out and total and specific IgE to respiratory and food allergens were determined through ImmunoCAP (Thermofischer, Sweden). Data were analyzed by descriptive statistics and results reported as median and interquartile range or mean and standard deviation. Categorical variables were reported as proportions. The study was approved by the local ethics committee (IRCCS San Matteo Hospital) and all patients provided written informed consent.

In our series of 14 adult patients (median age 41 years, IQR 25–48, M:F ratio 6:1; Table 1), food allergy was the second most frequent allergic comorbidity after allergic rhinitis, while sensitization to nsLTP was the most frequent (50%), followed by PR-10 (21.3%) and profilin (14.2%). Of note, most patients sensitized to peach displayed clinical symptoms after eating the peel of this fruit, ranging from contact dermatitis and oral allergic syndrome to anaphylaxis, and thus avoided eating peaches (Table 2). Besides, most patients reacted to other processed nsLTP containing foods, thus supporting the main clinical significance of the sensitization to this allergen as compared to thermolabile allergens, such as PR-10 and profilin. Additionally, sensitization to nsLTP was also present in patients with non-EoE EGID and associated with symptoms when eating a peach.

**Table 1:** Main demographic, clinical, and laboratory characteristics of the 14 included patients

Parameter	Result
Male sex, n (%)	12 (85.7)
Age, median (IQR; years)	41 (25–48)
Type of EGID	EoE (n = 6) EGE (n = 1) EC (n = 7)
Time since diagnosis, median (IQR; years)	EoE: 1 (0.25–1) EGE: 3 EC: 1.5 (1–4.5)
Tissue eosinophils at diagnosis, median (IQR; n/HPF)	EoE: 64 (30–95) EGE: 114 EC: 100 (100–107)
Blood eosinophils at diagnosis, median (IQR; cells/microL)	300 (180–330)
Serum ECP, median (IQR; U/mL)	28 (11.0–62.7) (11/14)
Main gastrointestinal symptoms at onset, n (%)	EoE: dysphagia, reflux, 4 (66.7) EGE: dyspepsia EC: abdominal pain, 5 (71.4)
Allergic comorbidities and sensitization profile	
Atopy^a^, n (%)	11 (78.5)
Allergic rhinitis, n (%)	10 (71.4)
Asthma, n (%)	2 (14.2)
Atopic dermatitis, n (%)	2 (14.2)
Food allergy, n (%)	5 (35.7)
Drug allergy, n (%)	2 (14.2)
Hymenoptera venom allergy, n (%)	1 (7.1)
PR-10^b^, n (%)	3 (21.3)
LTP^b^, n (%)	7 (50.0)
Profilin^b^, n (%)	2 (14.2)
IgE level, median (IQR; kU/L)	173 (112–417)

*EC* eosinophilic colitis, *ECP* eosinophilic cationic protein, *EoE* eosinophilic esophagitis, *EGE* eosinophilic gastritis, *HPF* high power field, *IQR* interquartile range, *LTP* lipid transfer protein, PR-10, pathogenesis-related-protein-10

^a^when at least one allergy was present

^b^assessed with specific IgE

**Table 2:** Sensitization and reactivity to food-containing non-specific lipid transfer protein (LTP) and other food in the seven patients sensitized to *Pru p3*

Patient #	EGID type	EGID therapy	*Pru p3* (kU/L)	*Ara h9* (kU/L)	*Jug r3* (kU/L)	*Cor a8* (kU/L)	*Tri a14* (kU/L)	*Bet v1* (kU/L)	Peach (symptoms)	Peanut (symptoms)	Nut (symptoms)	Hazelnut (symptoms)	Wheat (symptoms)	Other food causing symptoms	Peach avoidance
1)	EoE	PPI, 6-FED diet	10	4.3	9.8	3.8	1.4	neg	OAS, U	OAS	OAS	OAS, U	none	rice, grapes	Y
2)	EoE	PPI, swallowed fluticasone	8.16	5.2	1.3	2.4	neg	3.5	D	Diarrhea	none	none	none	soy, milk, banana	N
3)	EoE	6-FED	10.1	11.7	neg	neg	4.4	44.4	none	none	none	OAS	none	milk, egg, nuts, fennel, shrimp, soy	N
4)	EC	none	0.8	neg	neg	neg	neg	neg	U	none	none	none	none		Y
5)	EC	none	11.6	8.1	0.9	neg	neg	neg	OAS	A	OAS	none	none	apple, pear, cherry, kiwifruit	Y
6)	EC	enteric release budesonide	0.40	0.5	0.38	neg	neg	pos	A	OAS	OAS	none	none	none	Y
7)	EG	None	31.9	3.1	2.4	0.5	0.4	1.5	OAS	OAS	OAS	OAS	none	none	Y

*A* anaphylaxi, *D* dermatitis, *EC* eosinophilic colitis, *EG* eosinophilic gastritis, *EoE* eosinophilic esophagitis, *N* no, *OAS* oral allergic syndrome, *PPI* proton pump inhibitor, *U* urticaria, *Y* yes, *6-FED* 6-food elimination diet

^*^negative if specific IgE < 0.35 kU/L

To the best of our knowledge, this is the first study describing the sensitization to nsLTP in primary EGID adult patients. In our Italian cohort, the sensitization to nsLTP seems to be particularly relevant, and much greater than the rate of sensitization to this class of allergens in patients evaluated in allergology clinics in the Mediterranean area according to previous studies, which is estimated at about 9–12.3%, depending on the assay of detection [5]. Notably, a previous study from Spain has evaluated the sensitization to nsLTPs in EoE with specific IgE detection, by a combination of “singleplex” and “multiplex” assay or microarray, the latter to detect *nPru p3*, and estimated it a 20.9% [6]. The higher prevalence of sensitization to the peach nsLTP found in EoE patients in our study, as compared to that detected in the aforementioned one, may be related to the different assay used. More precisely, in our study a quantitative singleplex assay was used to detect *rPru p3*, whereas a semiquantitative microarray method was adopted to detect *nPru p3 *[6].

Fruits (mainly in the peel) and vegetables are the main source of nsLTP, and particularly peach with *Pru p3*, which is considered the primary sensitizer to this class of allergens in the Mediterranean area, though they are not usually part of the list of the commonly implicated allergens [7] and are not included in the “standard” food elimination diets for EoE treatment [3].

This study suggests that local epidemiology could influence the sensitization profile in primary EGID patients and this in turn could portend therapeutical implications as far as allergen avoidance strategies are concerned, at least in EoE. Indeed, the only allergen sources containing nsLTP which are at present considered in food elimination diets for EoE, such as the 6-food elimination diet (6-FED), are nuts and wheat. However, their avoidance may be of reduced or uncertain clinical benefit in EGID patients sensitized to *Pru p3*, because the quantity of nsLTP in nuts (such as nut and hazelnut) is lower compared to peach [8], and the degree of cross-reactivity of the LTP of wheat*, Tri a14,* with *Pru p3* is much lower [8, 9].

Due to their thermal and proteolytic resistance [5], nsLTPs may play a role as undenatured allergens in primary EGID, particularly with reference to esophagus, stomach, and proximal small bowel, as attested by experimental models of gastric and pancreatic digestion [5]. Their pathogenic role in other portions of the intestinal tract, and hence in eosinophilic colitis, is at present uncertain and needs further investigation. To conclude, peach avoidance should be considered according to the clinical history, in a subset of patients with primary EGID, especially in the Mediterranean area, and possibly combined to other allergen-free diets, according to the clinical characteristics of the patient.

## Data Availability

All data pertaining the study have been presented in the manuscript. Additional data can be asked to the corresponding author.

## Abbreviations

EoE: Eosinophilic esophagitis
EGID: Eosinophilic gastrointestinal disorder
nsLTP: Non-specific lipid transfer protein
